# Mutations in the UBIAD1 Gene, Encoding a Potential Prenyltransferase, Are Causal for Schnyder Crystalline Corneal Dystrophy

**DOI:** 10.1371/journal.pone.0000685

**Published:** 2007-08-01

**Authors:** Andrew Orr, Marie-Pierre Dubé, Julien Marcadier, Haiyan Jiang, Antonio Federico, Stanley George, Christopher Seamone, David Andrews, Paul Dubord, Simon Holland, Sylvie Provost, Vanessa Mongrain, Susan Evans, Brent Higgins, Sharen Bowman, Duane Guernsey, Mark Samuels

**Affiliations:** 1 Department of Ophthalmology and Visual Sciences, Dalhousie University, Halifax, Nova Scotia, Canada; 2 Department of Pathology, Dalhousie University, Halifax, Nova Scotia, Canada; 3 Montreal Heart Institute, University of Montreal, Montreal, Quebec, Canada; 4 Faculty of Medicine, Dalhousie University, Halifax, Nova Scotia, Canada; 5 Dipartimento di Scienze Neurologiche e del Comportamento, Università degli Studi di Siena, Siena, Italy; 6 Department of Ophthalmology, Faculty of Medicine, University of British Columbia, Vancouver, British Columbia, Canada; 7 Genome Atlantic, National Research Council of Canada Institute of Marine Biology, Halifax, Nova Scotia, Canada; 8 Department of Medicine, University of Montreal, Montreal, Quebec, Canada; Innsbruck Medical University, Austria

## Abstract

Schnyder crystalline corneal dystrophy (SCCD, MIM 121800) is a rare autosomal dominant disease characterized by progressive opacification of the cornea resulting from the local accumulation of lipids, and associated in some cases with systemic dyslipidemia. Although previous studies of the genetics of SCCD have localized the defective gene to a 1.58 Mbp interval on chromosome 1p, exhaustive sequencing of positional candidate genes has thus far failed to reveal causal mutations. We have ascertained a large multigenerational family in Nova Scotia affected with SCCD in which we have confirmed linkage to the same general area of chromosome 1. Intensive fine mapping in our family revealed a 1.3 Mbp candidate interval overlapping that previously reported. Sequencing of genes in our interval led to the identification of five putative causal mutations in gene UBIAD1, in our family as well as in four other small families of various geographic origins. UBIAD1 encodes a potential prenyltransferase, and is reported to interact physically with apolipoprotein E. UBIAD1 may play a direct role in intracellular cholesterol biochemistry, or may prenylate other proteins regulating cholesterol transport and storage.

## Introduction

**Figure 1:**
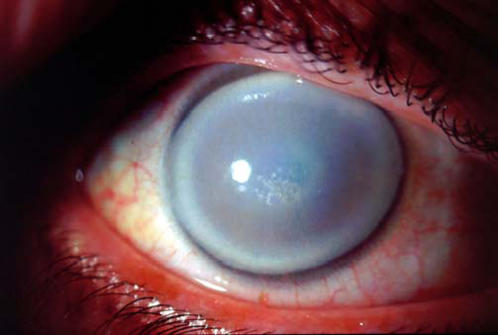
Slit lamp image of the right cornea from a 50-year old affected member of family 105, demonstrating a “bull's eye” morphology of central and peripheral corneal clouding associated with a relatively spared mid-peripheral zone. Central subepithelial crystalline deposits and prominent corneal arcus are also present.

Schnyder crystalline corneal dystrophy (SCCD, MIM 121800) is an inherited disorder whose most prominent feature is progressive, symmetrical opacification of the central cornea, the transparent anterior face of the eye (Fig. 1). Described first in 1924 by van Went and Wibaut[1], and later in more detail by Schnyder[2], SCCD is very rare. Until recently, the world literature contained fewer than 100 cases[3]. SCCD affects both sexes equally, and is found in multiple ethnic groups around the globe.

SCCD can become manifest as early as in the first few years of life, although it more commonly presents in the second decade. Thereafter, the clinical course is somewhat variable, although surprisingly good vision can be retained long-term despite significant corneal clouding[4]. Eventually however, reduced visual acuity and glare often mandate intervention. While phototherapeutic keratectomy (removal of superficial corneal layers via excimer laser ablation) can provide temporary relief in selected cases[5], the definitive treatment is surgical replacement of the central cornea (penetrating keratoplasty) with cadaveric donor tissue. SCCD can recur in the corneal graft postoperatively[4].

Pathophysiologically, SCCD appears to result from an abnormality in lipid metabolism in the cells of the cornea[6]–[14]. Examination of corneal tissue removed from affected patients during transplantation surgery has revealed a tenfold increase in mainly unesterified cholesterol levels, and a five- to ninefold increase in phospholipids[11], [12]. Immunohistochemical analysis of the same tissue is consistent with an underlying defect in HDL metabolism[11]. Although not a constant finding[7], SCCD has been associated in some patients with systemic dyslipidemia[9], [15]–[18] and thus possibly to an elevated risk of cardiovascular events such as myocardial infarction (heart attack) and stroke[9].

SCCD is inherited as an autosomal dominant trait with age-dependent penetrance, in which it is possible to assign affection status unambiguously by 40 years of age[19]. Although strongly genetic, identification of a causal gene has been elusive. Shearman *et al.* performed linkage analysis on a large family originally of Swedish/Finnish ancestry, localizing the defective gene to the short arm of chromosome 1, at 1p34–36[20], [21]. Theendakara *et al.* further refined the SCCD locus using families of multiple ethnicities, reducing the candidate region to a 2.32 Mbp (million base pair) interval lying between genetic markers D1S1160 and D1S1635, or possibly a smaller 1.58 Mbp interval between D1S503 and D1S1635[22]. Recently Aldave *et al.* and Oleynikov *et al.* have reported sequencing of all annotated genes within the larger interval, finding no pathogenic mutations and tentatively excluding them as causing SCCD[23], [24], a finding proposed to result from locus heterogeneity, mutations within promoter or untranslated regions, or the presence of an unannotated gene.

## Results

### Confirmation of linkage to chromosome 1p

**Figure 2:**
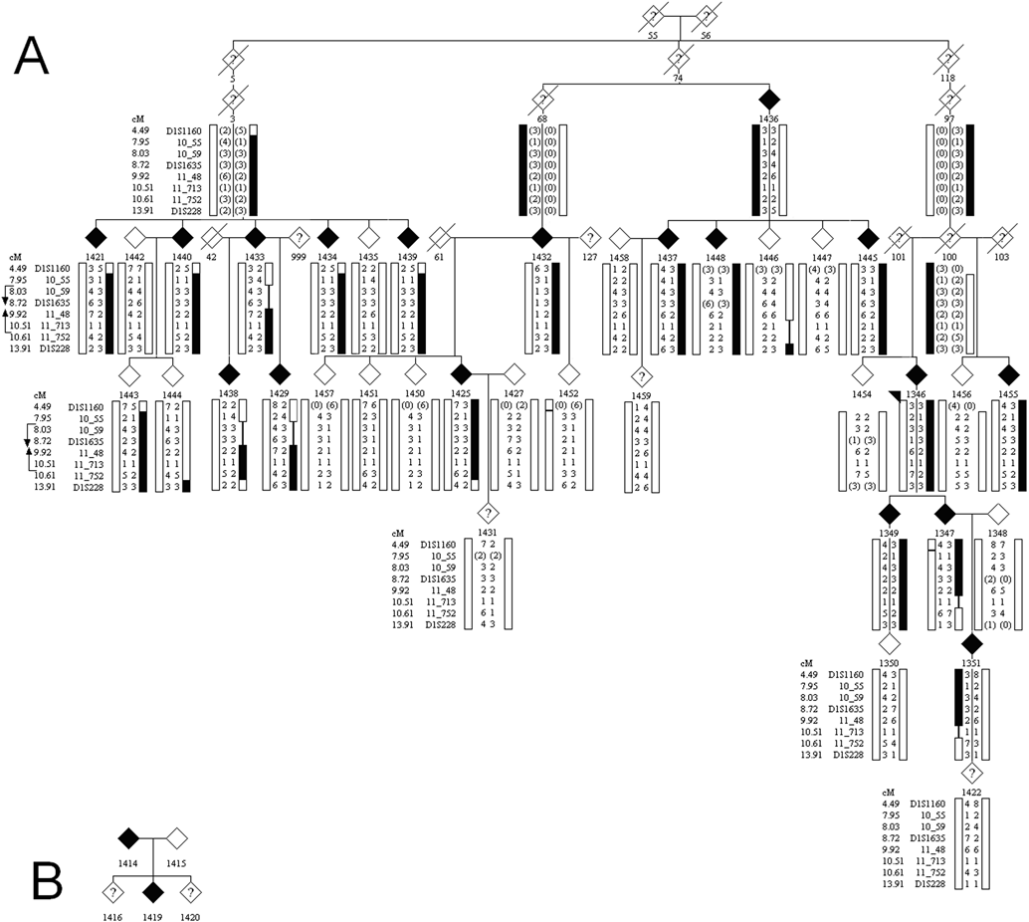
SCCD pedigrees. a) Family F105, showing affection status (filled symbols) and phased haplotypes generated by Simwalk for selected markers in the linked chromosomal region. Flanking recombinants in markers 10_55 and 11_752 are indicated. Genders are anonymized to preserve patient confidentiality. b) Family F115, showing affection status (filled symbols).

**Figure 3:**
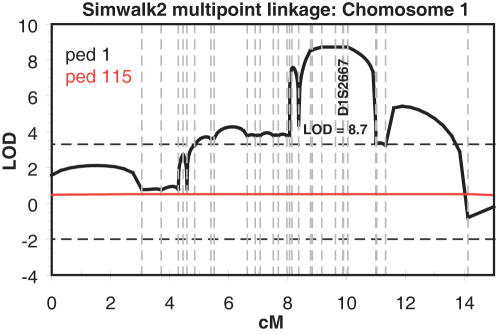
Multipoint linkage analysis using Simwalk for Family F105 (ped 1 in the figure) and F115 (ped 115 in the figure), across the linked chromosomal interval. In this analysis individual 1443 of family 105 was set as phenotype unaffected; the maxLOD increased slightly to 9.5 when 1443 was set to phenotype unknown. A maxLOD = 6.6 was obtained using an affecteds-only model.

Initial microsatellite genotyping using selected markers from the published linkage interval[22] on chromosome 1 were consistent with linkage in family F105 and F115 (see Materials and Methods and Fig. 2 for ascertainment and descriptions of families segregating SCCD). The two families together generated a multipoint sumLOD score of 8.7 using the 90% penetrance transmission model, with family F105 providing essentially all the statistical power (Fig. 3).

### Fine mapping the SCCD locus

We then performed extensive fine mapping utilizing 45 additional microsatellite markers from published databases or novel markers developed in our laboratory. All affected individuals segregated a shared haplotype in family F105 (simplified haplotype shown in Fig. 2). Recombinant haplotypes in affected individuals 1433 and 1347 (and inherited in each case by an affected child) defined a likely interval from marker 10_55 to 11_752 (corresponding to physical map locations of 10.55 Mbp and 11.752 Mbp, respectively) containing the causal gene. The haplotype shared *de facto* by the two affected individuals in F115 did not further reduce this interval, which comprises approximately 1.3 Mbp according to build 36, and contains 20 annotated genes in public databases.

Although there is no known genealogical relationship between the families F105 and F115, the affected haplotypes in the two families shared a small region of three consecutive markers (10_59, 10_70, 10_89) identical by state. These markers defined a potentially shared interval of 361 Kbp containing three genes (3′ end of PEX14, CASZ1, SRG [not currently annotated by RefSeq]).

### Mutation detection of SCCD

Mutation detection by direct DNA sequencing was initiated in the small potentially shared interval between these two families. However, no obviously causal mutations were identified in any of the three genes; moreover several SNPs were detected which segregated differently in the two families, suggesting that the microsatellite allele sharing by state was coincidental and not reflective of identity by descent. Therefore we extended sequencing of coding exons across the entire 1.3 Mbp interval defined in family F105.

Following tentative prioritization based on known or predicted biological function, all or part of 6 additional genes (C1ORF127, KIAA1337, MASP2, ANGPTL7, FRAP1 and UBIAD1) were sequenced. Approximately 125 distinct coding exons were sequenced in total, when potentially causal variants were detected in both families in gene UBIAD1. In family F105, a heterozygous missense variant c.355A>G (p. Arg119Gly) was identified (see Figure S1 for all sequence traces), which segregated to all 18 affected individuals in the pedigree consistently with being on the affected haplotype. In family F115, a heterozygous missense variant c.524C>T (p.Thr175Ile) was identified, which segregated to the two affected individuals in the pedigree. Neither variant was detected in any sampled unaffected individuals in the pedigrees, with the possible exception of individual 1443 whose phenotypic status is uncertain (see Materials and Methods), nor in 144 control samples (288 chromosomes) collected from the general Nova Scotia population, nor in 59 unrelated Caucasian CEPH HapMap DNA samples (118 chromosomes), nor in 89 unrelated Asian HapMap DNA samples (178 chromosomes). Neither variant occurs in dbSNP.

**Figure 4:**
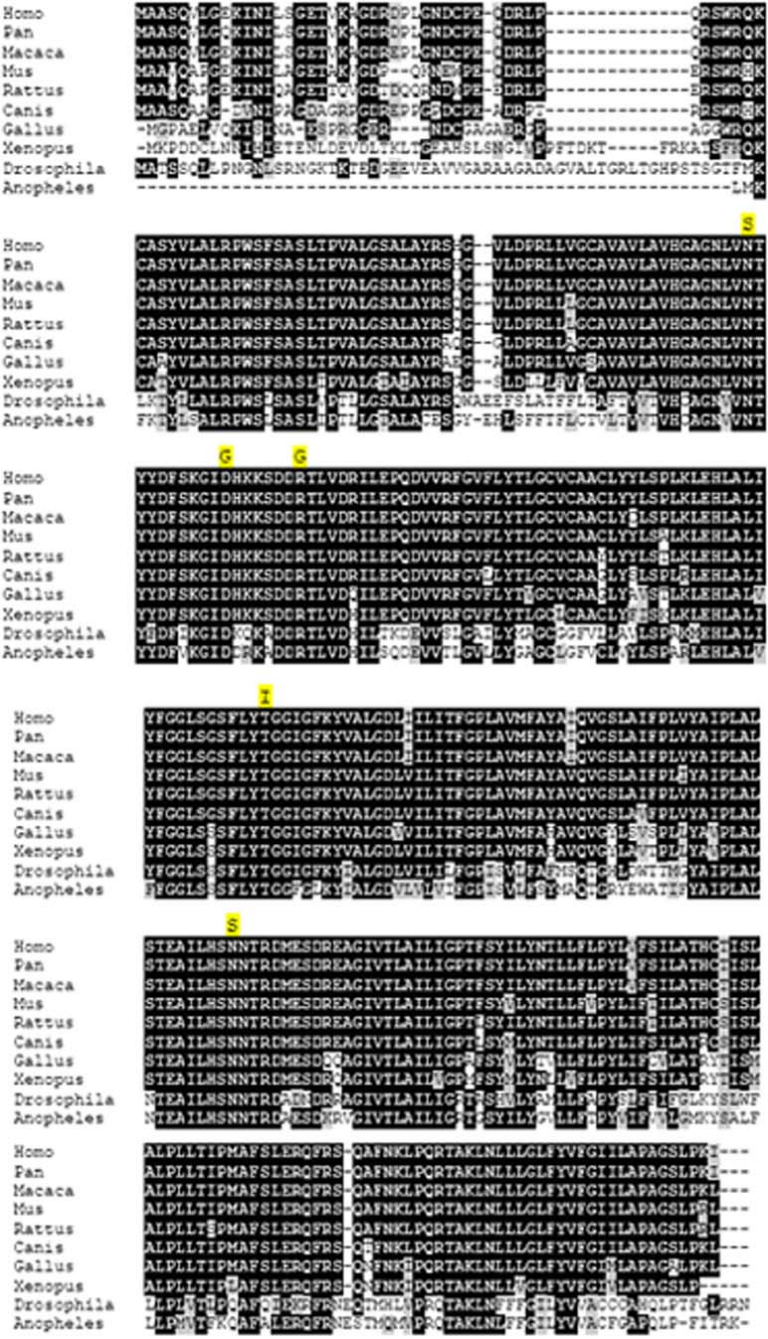
ClustalW alignment of vertebrate and invertebrate UBIAD1 putative orthologs. The five familial mutations are highlighted in yellow above the mutated residue.

Sequencing of UBIAD1 for affected patients from the three remaining families detected additional heterozygous missense variants in each of these pedigrees, c.695A>G (p.Asn232Ser) in F118, c.335A>G (p.Asp112Gly) in F122, and c.305A>G (p.Asn102Ser) in F123, likewise in residues identical across vertebrates and invertebrates (Fig. 4). None of the variants was detected in the Nova Scotia or HapMap control DNA samples or in dbSNP. Three Nova Scotia and four Caucasian CEPH HapMap control samples contained a heterozygous missense variant, c.224C>T (p.Ser75Phe), which appears to be a moderately common (approximately 3%) polymorphism in Caucasian populations. The identification of five different segregating, rare missense variants in an extremely conserved gene, strongly supports the identification of UBIAD1 as the causal gene for Schnyder crystalline corneal dystrophy. The data suggest limited if any genetic heterogeneity for this phenotype.

Public databases report three UBIAD1 transcripts of 1.5, 3.1 and 3.5 kb. The 1.5 kb transcript is attributable to the 1520 bp UBIAD1 reference sequence in NCBI (NM_013319.1, or CCDS129.1) and Ensembl (Build 38) coding for a 338 amino acid protein. The 3.1 kb Ensembl (Build 38) gene prediction corresponds to ENST00000240179 and NCBI cDNA clone AK074890. The 3.5 kb transcript corresponds to the Ensembl (Build 38) gene prediction ENST00000376810. These transcript variants all encode the same 338 amino acid protein product that was screened by our sequence analysis. There is a rare isoform variant that is predicted to splice out the UBIAD1 second exon and add three additional amino acids to the 3′end of exon 1 (Ensembl ENST00000376804; Expasy Q9Y5Z9-2). These additional 3 amino acids are derived from a putative ubiguitin-conjugating enzyme E2 variant 2 (UBE2V2) pseudogene that is approximately 8.6 kb from the 3′ UBIAD1 second exon (NCBI Accession AL031291), suggesting that this derives from an aberrantly spliced message.

### Bioinformatics analysis of UBIAD1

UBIAD1 is a highly conserved gene, almost 100% identical across much of its length in vertebrate genomes and with extensive homology in insects. All five putative causal variants detected in families with SCCD occur at amino acid residues which are identical in mammalian, avian, fish, and insect putative orthologs (Fig. 4).

**Figure 5:**
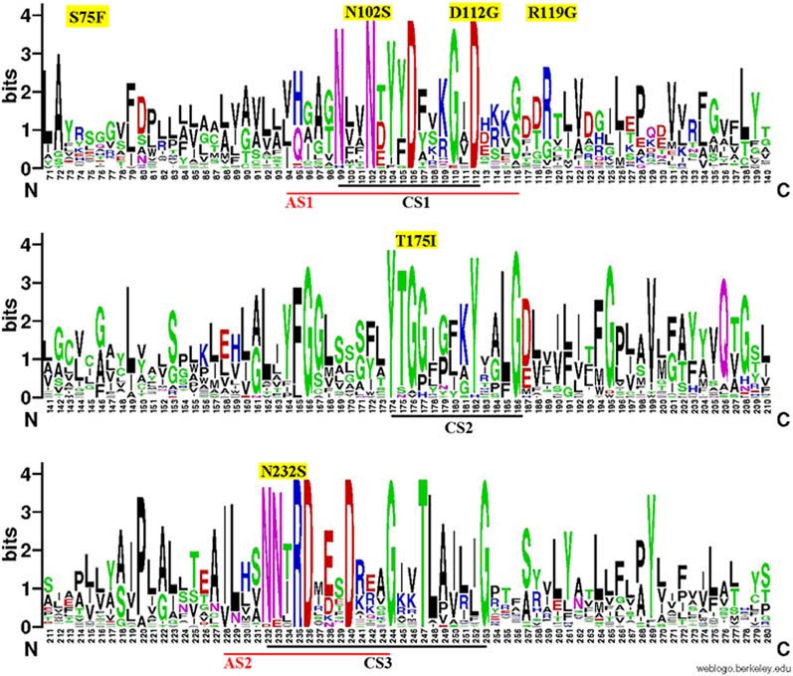
Conserved amino acid residues in three regions of UBIAD1 containing familial mutations (CS1, CS2, CS3). Also shown are two regions aligning with putative bacterial UbiA active sites (AS1, AS2), which are overlapped with CS1 and CS3, respectively. Familial mutations plus the control variant detected in this study are highlighted in yellow above each consensus plot. The sequence logo was generated with the multiple sequence alignment of distant orthologs selected from Eukaryota, Bacteria, and Archaea. The pairwise alignment of human UBIAD1 and *E.coli* UbiA peptide sequences aligned by ClustalW was used to annotate the regions of putative active sites.

InterPro, Pfam and ProSite all predict that UBIAD1 contains a prenyltransferase domain from residues 58-333, for which the archetype is bacterial protein UbiA (hence the name, UBIAD1). All five detected familial mutations occur in this domain (Fig. 5). PSORTII predicts 7 transmembrane domains and an integral membrane localization. No signal peptide or cleavage signal is predicted by SignalP. No prenylation sites were predicted by PrePS.

Three tools, SIFT, PANTHER and POLYPHEN were employed to judge the potential pathogenicity of the five familial plus one control missense variant. The results of prediction are shown in Table 1. The familial variants are predicted to have pathogenic consequences on the protein whereas the control variant p.Ser75Phe is predicted to be benign. All three methods predicted the familial mutations p.Asp112Gly and p.Thr175Ile have deleterious effects on protein function. Two out of three methods predicted other three familial variants p.Asn102Ser, p.Arg119Gly, p.Asn232Ser have damaging effects.

**Table 1:** Effects of mutations predicted by SIFT, PANTHER and PolyPhen.

Variants	Method		
	SIFT	PANTHER	PolyPhen
p.Ser75Phe	−	−	−
p.Asn102Ser	+	+	−
p.Asp112Gly	+	+	+
p. Arg119Gly	+	−	+
p.Thr175Ile	+	+	+
p.Asn232Ser	+	−	+

Sequence homology for SIFT prediction was calculated with the alignment of orthologs selected from Eukaryota. ‘−’ and ‘+’ indicate the predicted benign and deleterious effects of the mutations, respectively.

**Figure 6:**
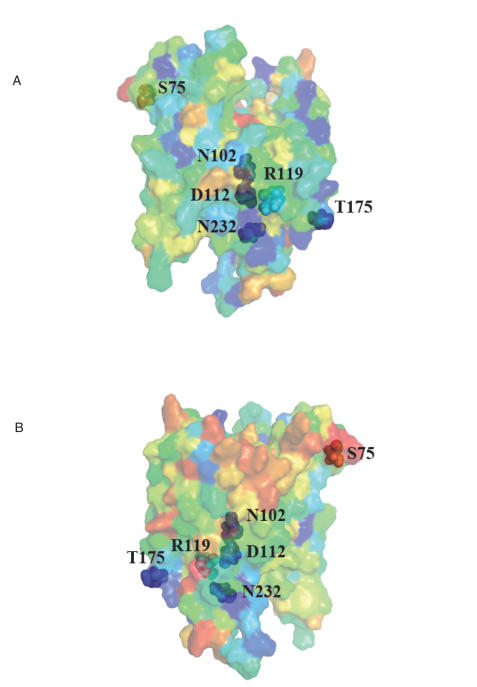
Predicted UBIA prenyltransferase domain-containing protein 1 structure from ModBase mapped with evolutionary conservation scores calculated by ConSurf. Five familial mutations plus one control variant detected in this study are indicated. The color scale ranging from blue to red represents the conservation score of residue varies from 9 (most conserved) to 1 (most variable). a, Front view; b, Rear view.

The evolutionary conservation score for each residue of UBIAD1 was calculated and mapped to a predicted 3-dimensional protein structure by ConSurf (Fig. 6). The scores for the 5 residues with familial missense mutations, p.Asn102Ser, p.Asp112Gly, p.Arg119Gly, p.Thr175Ile, and p.Asn232Ser are 9, 9, 7, 9, and 9, respectively. The score for control polymorphism p.Ser75Phe is 1. The familial variants also lie close to each other in contrast to the control variant on the predicted protein structure model. Structurally and functionally important regions in the protein typically appear as patches of evolutionarily conserved residues that are spatially close to each other[25]. The evolutionary conservation and the physical proximities of the five familial variants support that the variants are in a functional region of UBIAD1 protein.

## Discussion

We have identified the putative causal gene for Schnyder crystalline corneal dystrophy (SCCD) through a positional candidate strategy. We ascertained a large Nova Scotia family, as well as four small families, segregating SCCD in a dominant transmission pattern. Within the large family we were able to confirm linkage to the published locus at chromosome 1p36.2–36.3 with high statistical significance (maxLOD = 8.7), and we defined a minimal recombinant interval containing 20 annotated genes. Direct DNA resequencing of coding exons in the region identified five different heterozygous mutations in the gene UBIAD1, one mutation in each family. Each mutation was carried by all affected individuals within the respective family, and none was found in 144 unaffected control DNA samples (288 chromosomes) from the Nova Scotia population, nor 59 Caucasian HapMap DNA samples (118 chromosomes), nor 89 Asian HapMap DNA samples (178 chromosomes), nor are any of these mutations found in the dbSNP database. With one possible case of incomplete penetrance, sampled unaffecteds did not carry any of these mutations. All five mutations are in highly conserved residues in putative gene orthologs from other vertebrate (chimp, macaque, dog, mouse, rat, chicken, clawed toad) and invertebrate (*Drosophila*, *Anopheles*) genomes.

The recombinant interval defined by our family 105 overlaps with, but is offset slightly centromeric to that previously defined[22]. The location of gene UBIAD1 itself is consistent with chromosomal haplotypes in 70 of the 71 affected individuals from 13 families described in the previous study, with the exception of one recombinant individual, affected III:4 in family 9. The source of the inconsistency is unclear, with potential explanations including genetic heterogeneity, phenotypic misdiagnosis, microsatellite marker mutation or other technical difficulty with genotyping. Interestingly, a 343 kb copy number variant (CNV) has been annotated to occur in the genomic region including marker D1S1635[26], which if found in the key recombinant individual could have led to misleading definition of the recombinant boundary. Further suggestion of this possibility is that Theendakara *et al.* document segregation of two alleles for marker D1S3153, which however does not actually contain a microsatellite repeat but is also within the potential CNV region. In any case, direct resequencing of UBIAD1 has not been reported by other groups working on the genetics of SCCD, hence there is no inconsistency between our results and other published work at the nucleotide level.

Bioinformatics packages overall agreed in predicting likely pathogenicity for the five familial mutations, but less so for a missense variant identified in seven control samples. While UBIAD1 is ubiquitously expressed[27], the eye has been identified to have the highest normalized expression distribution of the 39 tissues reported at the Source (http://genome-www5.stanford.edu/ ) and of the 47 tissues identified at the Unigene Expression Profile Viewer (http://www.ncbi.nlm.nih.gov/UniGene/ESTProfileViewer.cgi?uglist = Hs.522933 ).

Although little has been known until now about UBIAD1 from the perspective of vertebrate genetics, the primary amino acid sequence is tantalizing. Bioinformatics analysis suggests that this gene is an intrinsic membrane protein with a prenyltransferase functional domain. UbiA, the canonical family member, also known as 4-hydroxybenzoate octaprenyl transferase, catalyzes 1,4-dihydroxy-2-naphthoate –>dimethylmenaquinone, in the ubiquinone biosynthetic pathway of bacteria (not to be confused with the UbiA gene in *C. elegans* which encodes ubiquitin and has no sequence homology to *E. coli* UbiA or UBIAD1). Although there is extensive sequence divergence between human UBIAD1 and *E. coli* UbiA, nonetheless the sequences can be aligned. Direct conservation of mutated residues in our families is not evident across such a large evolutionary divide, but four of the five familial variants we detected lie near or within predicted active site regions of the bacterial enzyme based on molecular modeling and multigenome alignment (Fig. 5)[28]. This supports, albeit indirectly, a deleterious effect of mutations in these regions of the protein. UBIAD1 need not be a true enzymatic prenyltransferase, it might simply contain ligand binding pockets for related molecules. Interestingly no prenylation sites were predicted for UBIAD1 by PrePS, suggesting that UBIAD1 is probably not self-modifying. However, the same result was found for UbiA of *E. coli* K12, indicating that prenylation sites are not automatically found in prenyltransferases themselves. PrePS did correctly predict farnesyltransferase and geranylgeranyltransferase modification sites for human c-K-ras2 protein isoform a.

The role of prenyltransferases or even prenyl binding proteins in lipid or cholesterol metabolism can be imagined. It seems unlikely that UBIAD1 plays a direct metabolic role in cholesterol biosynthesis. However, prenyl binding proteins such as UBIAD1 might play a role in sensing and regulating metabolite levels intracellularly and/or systemically. UBIAD1 might prenylate other proteins thereby influencing their intracellular localization. It is noteworthy that corneal lipid deposition is observed in three other human genetic disorders of cholesterol, specifically high density lipoprotein (HDL), metabolism: Niemann-Pick type C[29], LCAT deficiency (fish-eye disease) and ABCA1/Tangier Disease (TD)[11]. It is also suggestive that UBIAD1 has been shown to bind directly to apolipoprotein E, a component of very low density lipoprotein particles, in protein-protein interaction studies[30]. It is intriguing to speculate that UBIAD1 may play a role in cardiovascular disease, or may be a potential novel target for modulation of circulating or intracellular HDL cholesterol levels. As a significant caveat to our interpretation, biochemical studies will be required to verify the predicted prenyl binding and/or prenylation activities of UBIAD1. Measurements of circulating lipid particles (including HDL subtypes) and perhaps particle flux in affected individuals would also be informative, although these have not yet been attempted in our patients. Direct studies of HDL particle assembly in cell culture models may also clarify the role of UBIAD1.

Unexpectedly, UBIAD1 also has a proposed role in cancer. The gene was detected in gene expression studies in transient bladder carcinoma cells, and named TERE1[27], [31]. It is upregulated in particular cancer types. Clearly a directed study of intracellular cholesterol transport in the relevant cancers may clarify the role of TERE1/UBIAD1. Since cell growth is strongly dependent on synthesis of novel membrane components, pharmaceutical inhibition of UBIAD1 could potentially lead to reduction of unrestricted cell growth.

Mutations are known in the putative Drosophila UBIAD1 ortholog *heixuedian* (*heix*)[32]. These exhibit an array of cellular and developmental phenotypes including abnormal imaginal disc growth, hemocyte overgrowth and melanotic tumors, and wing abnormalities. Other than P-element insertions, the molecular bases of *heix* alleles have not been reported, nor have subcellular histological examinations been reported. It will be interesting to examine *heix* mutants in the light of our results, to determine whether abnormal lipid transport or intracellular cholesterol deposition underlie the developmental defects.

## Materials and Methods

Ethical approval for this study was obtained from the Research Ethics Board of the Queen Elizabeth II Health Sciences Centre.

### Clinical Assessment

We ascertained a large family from Nova Scotia known on a longstanding basis to local corneal specialists. The family, F105, unilineally segregates SCCD in 18 living affecteds and offered an excellent opportunity to discover the identity of the defective gene responsible for this disorder (Fig. 2a). This family is of uncertain, but possibly Spanish ancestry. Concurrently, we identified another nuclear family with SCCD, originally from Scotland, that had recently immigrated to Nova Scotia (F115, two affecteds, Fig. 2b). Subsequently, two further families were recruited from the clinical practices of Canadian corneal specialists: family F118, previously described elsewhere[12], containing two affected members of unknown ancestry, and family F122, of East Indian descent, with one known affected member. Lastly, family F123 with two available affected members, also previously described elsewhere[7], was recruited from colleagues in Italy.

Affection status of participants was determined in the following manner. Individuals were regarded as affected if typical corneal features of SCCD were present on slit lamp examination, or in documentary slit lamp images, or if corneal transplantation had been performed with SCCD as the underlying diagnosis. Individuals were regarded as unaffected if, by the age of forty, no features of SCCD were found. To facilitate phase determination during subsequent haplotype analysis, we collected DNA specimens from as many family members as possible. In the case of participants residing outside of Nova Scotia, in whom direct examination was not possible, individuals older than forty years of age without a definite diagnosis of SCCD and a history of normal routine eye examinations were considered to be probably unaffected. One individual, 1443 in family 105, is also of uncertain phenotype; visual exam did not indicate status as affected, however full slit-lamp examination could not be conducted due to local circumstances.

Control DNA samples were obtained from a randomized collection of apparently healthy individuals in the local Nova Scotia population, biased toward Caucasian ancestry. Additional controls were derived from the set of generally available HapMap DNA samples, specifically the 60 CEPH Caucasian parents and 90 East Asian unrelated individuals. Affection status of controls was unknown, but the possibility that any would be affected by SCCD seems remote, given its rarity and the likelihood in the case of the local population controls that they would have come to the attention of clinicians in the academic hospital system associated with Dalhousie University.

Following written informed consent, saliva or venous blood samples were obtained from all ascertained subjects, from which DNA was extracted according to standard protocols. We used the Oragene kits from DNA Genotek (Ottawa, Canada) for self-collection of salivary DNA (particularly in situations where it was desirable for samples to be collected via mail), with excellent DNA yields and performance during microsatellite genotyping and direct sequencing.

### Genotyping and Linkage Analysis

Microsatellite genotyping was performed using fluorescent primers. 5′ tags were added to the reverse, unlabelled primer in each case to reduce variable non-templated nucleotide addition[33], [34]. Products were resolved on ABI 377 electrophoresis instruments and genotype chromatograms were interpreted using the GeneMarker program from SoftGenetics, Inc.[35] See Table S1 for details of custom marker primers.

Pedigree files and genotype data were imported into Progeny Lab software version (6.6.01). Mendelian inconsistencies were identified with Pedcheck version 1.1[36]. Allele calls for inconsistent markers were set to 0 in the offending nuclear families involved in the inconsistencies. Genetic positions from the Decode map were used when available[37]. To calculate genetic position for markers not on the Decode map, linear interpolation was used between the two closest common markers flanking the markers to position, using physical distances provided by human genome assembly, build 36.

Statistical analyses were conducted with two models.

1. An affected only model in which all individuals not known to be affected except spouses were set to unknown and using penetrance set to 0.99, phenocopy rate set to 0.001 for a dominant disease with allele frequency of 0.001.
2. Penetrance set to 0.90 with a phenocopy rate of 0.001 and a dominant disease allele frequency of 0.001.

Marker allele frequencies were estimated by maximum likelihood using Merlin version 1.0.1 (option –fm)[38]. As Merlin can not handle large pedigrees, pedigree 105 was divided into three smaller families (branch 2/3, 74/75 and 100/103/101) for this stage of the analysis. Allelic frequencies from Merlin were manually incorporated into dat files.

Two-point linkage was carried out using the MLINK routine of FASTLINK v4.1P on Linux. LOD scores were compiled by extracting results from the final.out output file using MLINK_LODS v2. Multipoint linkage analysis and haplotyping were carried out using SIMWALK version 2.90 on Linux[39]. The input files were converted to SIMWALK format using Mega2 v3.0 R4. The haplotype routine converged on the first run for both pedigrees.

### Mutation Detection

Predicted protein coding regions of all examined genes were amplified using primers designed with Primer3[40] (http://frodo.wi.mit.edu/) (see Table S2 for sequences) from two affected individuals from family F105 and one affected individual from family F115. Coding exons of gene UBIAD1 were subsequently sequenced in samples from additional affected individuals in all five families, and from controls. PCR products were sequenced using ABI 377 or 3700 electrophoresis instruments at the Genome Atlantic and Institute for Marine Biology TAGC or at the McGill University and Genome Quebec Centre for Innovation. Sequence chromatograms were interpreted using the MutationSurveyor program from SoftGenetics, Inc., with gene annotations from GenBank[35].

### Bioinformatics Analysis

InterPro, Pfam, ProSite, PSORTII, SignalP, and PrePS were run via the Expasy web site (http://us.expasy.org/tools/ ). The effects of amino acid substitutions on protein function were predicted with SIFT[41]–[43], PolyPhen[44]–[46], and PANTHER[47], [48]. Homologous peptide sequences of human UBIAD1 gene in Eukaryota, Archaea and Bacteria were retrieved through NCBI web site using protein-protein BLAST (blastp) against the nr database. Multiple sequence alignments were computed by ClustalW and displayed with BoxShade. The sequences of distantly related orthologs were aligned by MUSCLE[49]. The sequence logo in Fig. 5 was created by WebLogo[50]. The evolutionary conservation of amino acid sites with mutations was analyzed using ConSurf[25], [51], [52], based on alignments shown in Fig. S2 and S3. The predicted protein structure from ModBase[53] for the UbiA prenyltransferase domain-containing protein 1 was used to build a 3D model. Figure 6 was generated using PyMOL[54].

## Supporting Information

Table S1 Custom microsatellite genotyping marker primer data.
(0.17 MB DOC)

Table S2 Primer sequences for mutation detection amplification of UBIAD1 coding exons (two amplicons for each exon).
(0.03 MB DOC)

Figure S1 Mutation detection sequencing traces for affected patients from each of the five families with SCCD, following fluorescent sequencing on ABI 377 or 3700 electrophoresis instruments and alignment to annotated genomic sequences containing the UBIAD1 gene using MutationSurveyor. Each panel has 7 lines generated by the software: from top to bottom are the amino acid translations of consensus and predicted mutation sequences, forward direction virtual reference trace, forward direction patient sequence trace, forward direction mutation call, reverse direction mutation call, reverse direction patient sequence trace, reverse direction virtual reference trace. a, Family F105; b, Family F115; c, Family F118; d, Family F122; e, Family F123.
(0.33 MB TIF)

Figure S2 Multiple sequence alignment of the Eukaryota orthologs of Human UBIAD1 peptide sequence. The alignment was used to study the sequence conservation and predict the effects of mutations.
(3.89 MB TIF)

Figure S3 Multiple sequence alignment of distant orthologs of Human UBIAD1 peptide sequence selected from Eukaryota, Bacteria, and Archaea. The alignment was used to study the sequence conservation and generate the sequence logo.
(4.73 MB TIF)
