# Supplementary material for: Mutations in the UBIAD1 Gene, Encoding a Potential Prenyltransferase, Are Causal for Schnyder Crystalline Corneal Dystrophy
Source: PLoS One. 2007 Aug 1;2(8):e685. doi: 10.1371/journal.pone.0000685 (PMC1925147; doi:10.1371/journal.pone.0000685)
Supplement: Table S1 — Custom microsatellite genotyping marker primer data. (0.17 MB DOC) [file pone.0000685.s001.doc]

| **Marker name** | **Position** | **Repeat** | **Copy #** | **For/Rev** | **Primers** | **Size** |
| --- | --- | --- | --- | --- | --- | --- |
| 7.51 | [chr1:7514527-7514589](http://genome.ucsc.edu/cgi-bin/hgTracks?hgsid=65143549&db=hg17&position=chr1%3A7514527-7514589) | CCT | 21 | **F** | FAM-CAACTCATTTCCAGGGCTTT | 241 |
| 7.51 | [chr1:7514527-7514589](http://genome.ucsc.edu/cgi-bin/hgTracks?hgsid=65143549&db=hg17&position=chr1%3A7514527-7514589) | CCT | 21 | **R** | GTTTCTTGTGGTCCATAGACCCCACAG |  |
| 7.9 | [chr1:7900726-7900836](http://genome.ucsc.edu/cgi-bin/hgTracks?hgsid=65127370&db=hg17&position=chr1%3A7900726-7900836) | AAGA | 27.5 | **F** | HEX-CACACCTTTCCTTCGTCCAT | 277 |
| 7.9 | [chr1:7900726-7900836](http://genome.ucsc.edu/cgi-bin/hgTracks?hgsid=65127370&db=hg17&position=chr1%3A7900726-7900836) | AAGA | 27.5 | **R** | GTTTCTTTTCAAGGGGGTAGTAGGGAGT |  |
| 8.37 | [chr1:8366834-8366869](http://genome.ucsc.edu/cgi-bin/hgTracks?hgsid=65127370&db=hg17&position=chr1%3A12201679-12201713) | GT | 18 | **F** | NED-AAGTCTGGACACTCCCCTGA | 299 |
| 8.37 | [chr1:8366834-8366869](http://genome.ucsc.edu/cgi-bin/hgTracks?hgsid=65127370&db=hg17&position=chr1%3A12201679-12201713) | GT | 18 | **R** | GTTTCTTTAAGGCCAGCAGTCCTCATC |  |
| 8.81 | chr1:8810155-8810197 | TG | 21.5 | **F** | FAM-TGGGAGAGAGAGAATGAATGTG | 132 |
| 8.81 | chr1:8810155-8810197 | TG | 21.5 | **R** | GTTTCTTGAGGTCGAAGGGAAAAGAGG |  |
| 9.25 | chr1:9245762-9245798 | TGT | 12.3 | **F** | HEX-TCTCCTTGCTGCCCTAGTTT | 151 |
| 9.25 | chr1:9245762-9245798 | TGT | 12.3 | **R** | GTTTCTTCTCCTTTCCCCCATGTCAG |  |
| 9.87 | chr1:9871170-9871214 | AC | 22.5 | **F** | NED-TTGGGGCAAATACAATGAAAA | 180 |
| 9.87 | chr1:9871170-9871214 | AC | 22.5 | **R** | GTTTCTTTTCCTCACCATCCTTTCCTG |  |
| 10.00 | [chr1:9996114-9996168](http://genome.ucsc.edu/cgi-bin/hgTracks?hgsid=66683174&db=hg17&position=chr1%3A9996114-9996168) | TATT | 13.2 | **F** | /5HEX/ACTAACTTGTCCCAGATTACTGTGT | 117 |
| 10.00 | [chr1:9996114-9996168](http://genome.ucsc.edu/cgi-bin/hgTracks?hgsid=66683174&db=hg17&position=chr1%3A9996114-9996168) | TATT | 13.2 | **R** | GTTTCTTGGCAACAAAGGGAGACTCTG |  |
| 10.08 | chr1:10085118-10085156 | TG | 19.5 | **F** | /56-FAM/CGGTGGAATTTAGAAGCCTATG | 145 |
| 10.08 | chr1:10085118-10085156 | TG | 19.5 | **R** | GTTTCTTCCTGAATGATGTTCCCTTTCA |  |
| 10.1 | chr1:10096301-10096326 | GT | 13 | **F** | FAM-TCGCAAGTAGAAGGTTTTGGA | 257 |
| 10.1 | chr1:10096301-10096326 | GT | 13 | **R** | GTTTCTTTGCACCACTAGGAGGCTACA |  |
| 10.21 | [chr1:10207215-10207256](http://genome.ucsc.edu/cgi-bin/hgTracks?hgsid=66683174&db=hg17&position=chr1%3A10207215-10207256) | TA | 21 | **F** | NED-GTGGGAGGATTGCTTGAGG | 180 |
| 10.21 | [chr1:10207215-10207256](http://genome.ucsc.edu/cgi-bin/hgTracks?hgsid=66683174&db=hg17&position=chr1%3A10207215-10207256) | TA | 21 | **R** | GTTTCTTTGCTTAGCAAAAGCTATCCAAA |  |
| 10.29 | [chr1:10294562-10294592](http://genome.ucsc.edu/cgi-bin/hgTracks?hgsid=66683174&db=hg17&position=chr1%3A10294562-10294592) | AAT | 10.3 | **F** | /5HEX/ACCTAGCAGGCGGAGGTT | 195 |
| 10.29 | [chr1:10294562-10294592](http://genome.ucsc.edu/cgi-bin/hgTracks?hgsid=66683174&db=hg17&position=chr1%3A10294562-10294592) | AAT | 10.3 | **R** | GTTTCTTTGGTGATACTAAAAACTGTATGCAAAG |  |
| 10.37 | [chr1:10371159-10371197](http://genome.ucsc.edu/cgi-bin/hgTracks?hgsid=66683174&db=hg17&position=chr1%3A10371159-10371197) | AC | 19.5 | **F** | /56-FAM/CATGTGGCCTAACAAAAGG | 214 |
| 10.37 | [chr1:10371159-10371197](http://genome.ucsc.edu/cgi-bin/hgTracks?hgsid=66683174&db=hg17&position=chr1%3A10371159-10371197) | AC | 19.5 | **R** | GTTTCTTAAAAACAAAGGTGCCTGGTG |  |
| 10.55 | chr1:10549960-10549990 | GT | 15.5 | **F** | NED-CAGGGAGCTCTGTGTTTGAA | 250 |
| 10.55 | chr1:10549960-10549990 | GT | 15.5 | **R** | GTTTCTTACCTAATGAACGGGCAACAG |  |
| 10.59 | chr1:10585959-10586000 | TG | 21 | **F** | HEX-AATCTCTGTTCCCCAGCAAC | 276 |
| 10.59 | chr1:10585959-10586000 | TG | 21 | **R** | GTTTCTTGGGCAGCCTGACATACCTAC |  |
| 10.70 | [chr1:10697269-10697309](http://genome.ucsc.edu/cgi-bin/hgTracks?hgsid=66683174&db=hg17&position=chr1%3A10697269-10697309) | GA | 20.5 | **F** | /5HEX/CGGGGAAATCCAATACGCTGAA | 270 |
| 10.70 | [chr1:10697269-10697309](http://genome.ucsc.edu/cgi-bin/hgTracks?hgsid=66683174&db=hg17&position=chr1%3A10697269-10697309) | GA | 20.5 | **R** | GTTTCTTCCGTCTCTCTTGCTGTCCTC |  |
| 10.78 | chr1:10777570-10777611 | GT | 21 | **F** | /56-FAM/ATGTCCGGGAATCAACACAC | 292 |
| 10.78 | chr1:10777570-10777611 | GT | 21 | **R** | GTTTCTTGAACTGCCCTGGAATGAACT |  |
| 10.86 | chr1:10860102-10860143 | GACA | 10.5 | **F** | NED-TCCCCAAAACTCTCTCCTCA | 292 |
| 10.86 | chr1:10860102-10860143 | GACA | 10.5 | **R** | GTTTCTTCAGGACCTCACAGCTCTTGG |  |
| 10.89 | chr1:10892343-10892370 | GT | 14 | **F** | /5HEX/AGGACCTGACCCTGAGACCT | 116 |
| 10.89 | chr1:10892343-10892370 | GT | 14 | **R** | GTTTCTTAGCTCTGAGCCATTCGAGAG |  |
| 10.96 | [chr1:10964417-10964502](http://genome.ucsc.edu/cgi-bin/hgTracks?hgsid=66683174&db=hg17&position=chr1%3A10964417-10964502) | GAAG | 21.8 | **F** | /56-FAM/TGTCTGTCCAACAAGAAGATGC | 145 |
| 10.96 | [chr1:10964417-10964502](http://genome.ucsc.edu/cgi-bin/hgTracks?hgsid=66683174&db=hg17&position=chr1%3A10964417-10964502) | GAAG | 21.8 | **R** | GTTTCTTATTGAAGCCAGGCTGAGAGG |  |
| 11.07 | chr1:11074887-11074928 | AC | 21 | **F** | FAM-TTCAGCATCATGTGGTTTGG | 145 |
| 11.07 | chr1:11074887-11074928 | AC | 21 | **R** | GTTTCTTTTTCCCCTATGTGACAGCATC |  |
| 11.29 | chr1:11286992-11287040 | AC | 24.5 | **F** | NED-TGTTGACTGTCTGGCCATCT | 180 |
| 11.29 | chr1:11286992-11287040 | AC | 24.5 | **R** | GTTTCTTAGGGCTCAGAGAGGAGCTGT |  |
| 11.41 | chr1:11407784-11407827 | AC | 22 | **F** | /5HEX/TTATCCCACCGCTTTCTCTG | 205 |
| 11.41 | chr1:11407784-11407827 | AC | 22 | **R** | GTTTCTTGAAATGGAGGAGGGGAAAAT |  |
| 11.48 | chr1:11475001-11475037 | CA | 18.5 | **F** | HEX-CAGACTCCCAAGCACAGACA | 164 |
| 11.48 | chr1:11475001-11475037 | CA | 18.5 | **R** | GTTTCTTGTCCCCTGGCAGGTGTAGTA |  |
| 11.483 | [chr1:11482961-11482991](http://genome.ucsc.edu/cgi-bin/hgTracks?hgsid=69933056&db=hg17&position=chr1%3A11482961-11482991) | TG | 15.5 | **F** | /5HEX/CCAGCGCTGTACCTAAGCTG | 121 |
| 11.483 | [chr1:11482961-11482991](http://genome.ucsc.edu/cgi-bin/hgTracks?hgsid=69933056&db=hg17&position=chr1%3A11482961-11482991) | TG | 15.5 | **R** | GTTTCTTCCACTTGGGTGTCTGTGCAT |  |
| 11.55 | chr1:11554335-11554368 | CA | 17 | **F** | /56-FAM/CAAACAAGACCCCAAACCAG | 230 |
| 11.55 | chr1:11554335-11554368 | CA | 17 | **R** | GTTTCTTGGGGGTGAGTAGCTCTTCTG |  |
| 11.554 | [chr1:11554335-11554368](http://genome.ucsc.edu/cgi-bin/hgTracks?hgsid=69933056&db=hg17&position=chr1%3A11554335-11554368) | CA | 17 | **F** | /56-FAM/CAAACAAGACCCCAAACCAG | 177 |
| 11.554 | [chr1:11554335-11554368](http://genome.ucsc.edu/cgi-bin/hgTracks?hgsid=69933056&db=hg17&position=chr1%3A11554335-11554368) | CA | 17 | **R** | GTTTCTTGCTCAGAGAGGGGTCTGAACT |  |
| 11.587 | [chr1:11586839-11586866](http://genome.ucsc.edu/cgi-bin/hgTracks?hgsid=69933056&db=hg17&position=chr1%3A11586839-11586866) | AAC | 9.3 | **F** | /5HEX/CCAGGACCTCCTGACTTGAC | 244 |
| 11.587 | [chr1:11586839-11586866](http://genome.ucsc.edu/cgi-bin/hgTracks?hgsid=69933056&db=hg17&position=chr1%3A11586839-11586866) | AAC | 9.3 | **R** | GTTTCTTCTGCTAGGCTGGATGCTACA |  |
| 11.685 | [chr1:11685247-11685284](http://genome.ucsc.edu/cgi-bin/hgTracks?hgsid=69933056&db=hg17&position=chr1%3A11685247-11685284) | GT | 19 | **F** | /56-FAM/AGACTCCCAGGGTCGTCAG | 298 |
| 11.685 | [chr1:11685247-11685284](http://genome.ucsc.edu/cgi-bin/hgTracks?hgsid=69933056&db=hg17&position=chr1%3A11685247-11685284) | GT | 19 | **R** | GTTTCTTGAGGTCGCTCCTGGATGTAG |  |
| 11.69 | chr1:11685247-11685284 | GT | 19 | **F** | NED-CGGTCTGAGAAGCTTCAGG | 253 |
| 11.69 | chr1:11685247-11685284 | GT | 19 | **R** | GTTTCTTCAGAAAGTGCGCAGAGTGG |  |
| 11.713 | [chr1:11713208-11713238](http://genome.ucsc.edu/cgi-bin/hgTracks?hgsid=69933056&db=hg17&position=chr1%3A11713208-11713238) | TTTG | 7.8 | **F** | /5HEX/TGGTTCTCATATACCTGCTTTGC | 136 |
| 11.713 | [chr1:11713208-11713238](http://genome.ucsc.edu/cgi-bin/hgTracks?hgsid=69933056&db=hg17&position=chr1%3A11713208-11713238) | TTTG | 7.8 | **R** | GTTTCTTGCTGGGGCGACAGAGCTA |  |
| 11.752 | [chr1:11752217-11752260](http://genome.ucsc.edu/cgi-bin/hgTracks?hgsid=69933056&db=hg17&position=chr1%3A11752217-11752260) | GT | 22 | **F** | /56-FAM/AGAAGTTTCGGTGAGCCAAG | 177 |
| 11.752 | [chr1:11752217-11752260](http://genome.ucsc.edu/cgi-bin/hgTracks?hgsid=69933056&db=hg17&position=chr1%3A11752217-11752260) | GT | 22 | **R** | GTTTCTTCTCCTCACTGGCTTGGAAAC |  |
| 11.807 | [chr1:11806988-11807034](http://genome.ucsc.edu/cgi-bin/hgTracks?hgsid=69933056&db=hg17&position=chr1%3A11806988-11807034) | TG | 23.5 | **F** | /56-FAM/ACCTTCAGCTTCGGTCTCCT | 292 |
| 11.807 | [chr1:11806988-11807034](http://genome.ucsc.edu/cgi-bin/hgTracks?hgsid=69933056&db=hg17&position=chr1%3A11806988-11807034) | TG | 23.5 | **R** | GTTTCTTGTGAGGGTGGAGAGTTCAGC |  |
| 11.85 | chr1:11855282-11855315 | TG | 17 | **F** | /5HEX/TGGGTGGGTAAGGGCTGTGTAA | 282 |
| 11.85 | chr1:11855282-11855315 | TG | 17 | **R** | GTTTCTTGGTGCTGGTTGATGAATCCT |  |
| 11.86 | chr1:11858376-11858415 | GT | 20 | **F** | NED-CACCTGCATAGGGCCATC | 186 |
| 11.86 | chr1:11858376-11858415 | GT | 20 | **R** | GTTTCTTCCCTCCCTCTGTTAACCATGT |  |
| 11.94 | chr1:11938746-11938777 | AC | 16 | **F** | /56-FAM/TGCTGGAGTTCAAGAGCCTGT | 306 |
| 11.94 | chr1:11938746-11938777 | AC | 16 | **R** | GTTTCTTGGCCTCACTACCTGAACCTG |  |
