# Supplementary material for: Mutations in the UBIAD1 Gene, Encoding a Potential Prenyltransferase, Are Causal for Schnyder Crystalline Corneal Dystrophy
Source: PLoS One. 2007 Aug 1;2(8):e685. doi: 10.1371/journal.pone.0000685 (PMC1925147; doi:10.1371/journal.pone.0000685)
Supplement: Table S2 — Primer sequences for mutation detection amplification of UBIAD1 coding exons (two amplicons for each exon). (0.03 MB DOC) [file pone.0000685.s002.doc]

| Exon 1 Left | F: CGG AAC CGA AGG AAG GTC |
| --- | --- |
|  | R: CCA AGA TTC GGT CCA CAA GT |
| Exon 1 Right | F: GGC TCT TGG TGG GTT GTG |
|  | R: AAA GCG GCT TAA ATT AGA AAG C |
| Exon 2 Left | F: AAG TGG CCT GCC TCT TCA C |
|  | R: GCT GAT GGT GCA GTG TGT G |
| Exon 2 Right | F: GGG AGG CTG GTA TCG TCA C |
|  | R: TGA CTG CCA AAT CAC ATT CC |
