# Supplementary figures and images for: Mutations in the UBIAD1 Gene, Encoding a Potential Prenyltransferase, Are Causal for Schnyder Crystalline Corneal Dystrophy
Source: PLoS One. 2007 Aug 1;2(8):e685. doi: 10.1371/journal.pone.0000685 (PMC1925147; doi:10.1371/journal.pone.0000685)

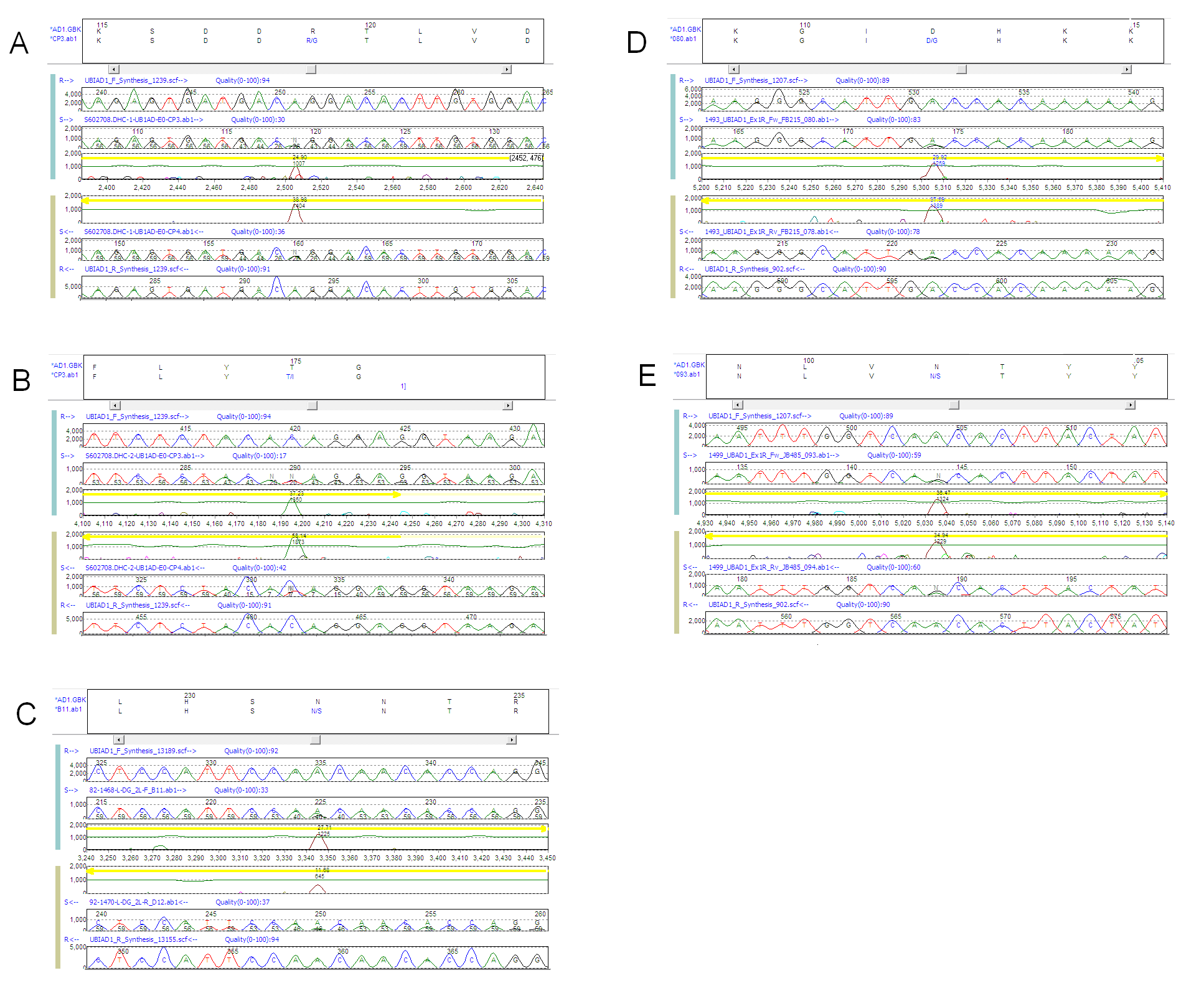

Supplement: Figure S1 — Mutation detection sequencing traces for affected patients from each of the five families with SCCD, following fluorescent sequencing on ABI 377 or 3700 electrophoresis instruments and alignment to annotated genomic sequences containing the UBIAD1 gene using MutationSurveyor. Each panel has 7 lines generated by the software: from top to bottom are the amino acid translations of consensus and predicted mutation sequences, forward direction virtual reference trace, forward direction patient sequence trace, forward direction mutation call, reverse direction mutation call, reverse direction patient sequence trace, reverse direction virtual reference trace. a, Family F105; b, Family F115; c, Family F118; d, Family F122; e, Family F123. (0.33 MB TIF) [file pone.0000685.s003.tif]

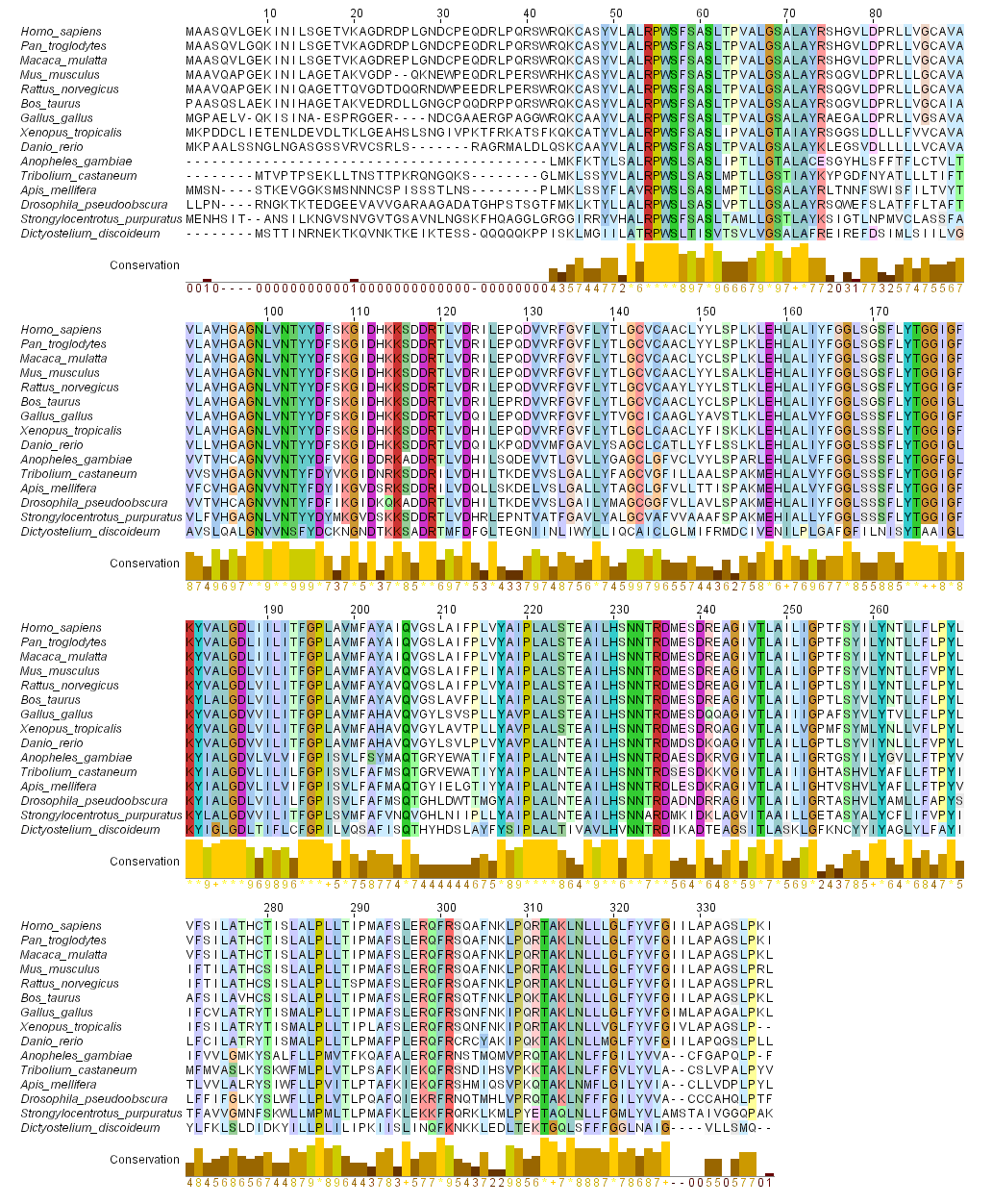

Supplement: Figure S2 — Multiple sequence alignment of the Eukaryota orthologs of Human UBIAD1 peptide sequence. The alignment was used to study the sequence conservation and predict the effects of mutations. (3.89 MB TIF) [file pone.0000685.s004.tif]

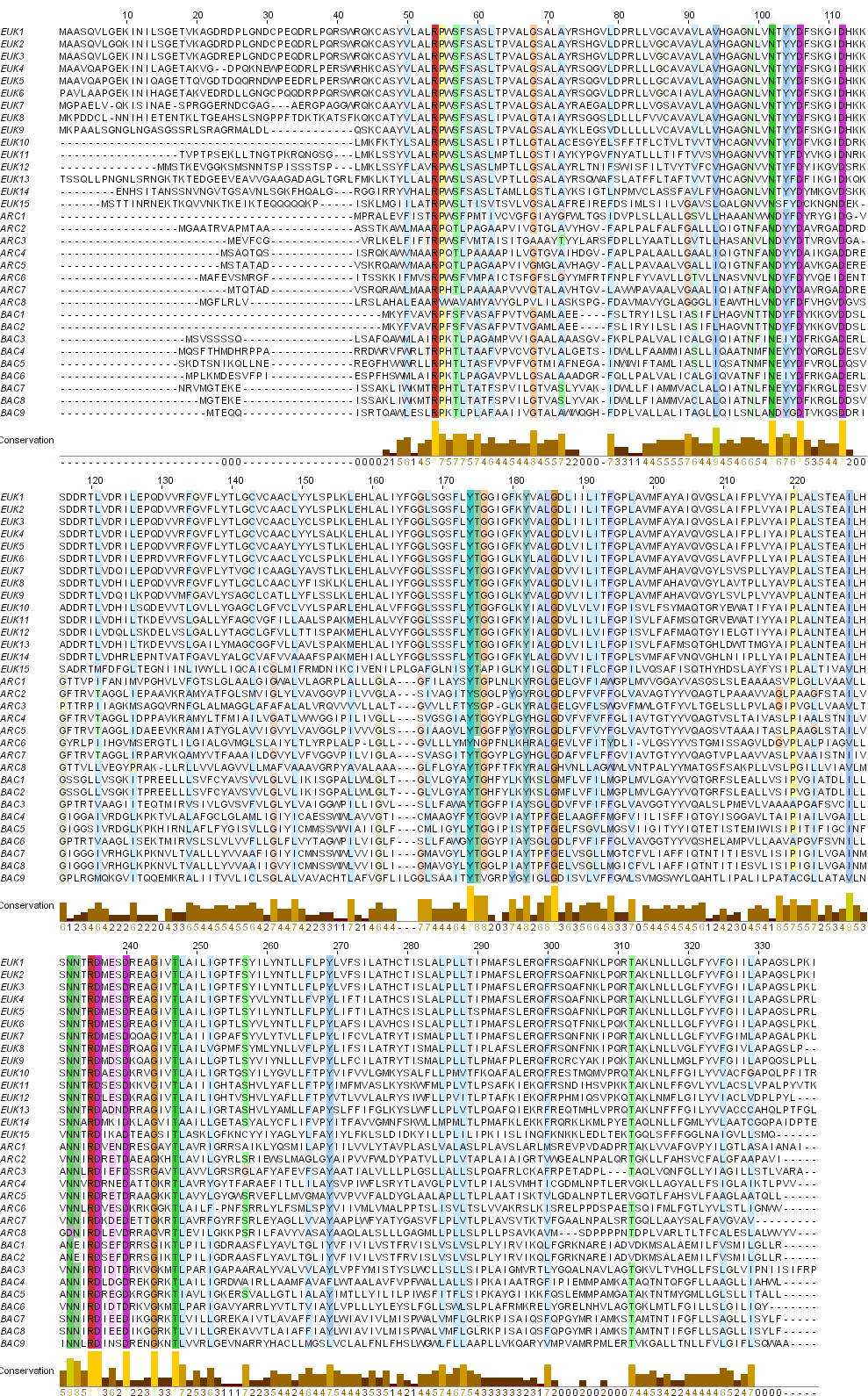

Supplement: Figure S3 — Multiple sequence alignment of distant orthologs of Human UBIAD1 peptide sequence selected from Eukaryota, Bacteria, and Archaea. The alignment was used to study the sequence conservation and generate the sequence logo. (4.73 MB TIF) [file pone.0000685.s005.tif]
